# Profile of COVID-19 in Brazil—risk factors and socioeconomic vulnerability associated with disease outcome: retrospective analysis of population-based registers

**DOI:** 10.1136/bmjgh-2022-009489

**Published:** 2022-12-14

**Authors:** Felipe A C Pereira, Fábio M H S Filho, Arthur R de Azevedo, Guilherme L de Oliveira, Renzo Flores-Ortiz, Luis Iván O Valencia, Moreno S Rodrigues, Pablo Ivan P Ramos, Nívea B da Silva, Juliane Fonseca de Oliveira

**Affiliations:** 1 Center for Data and Knowledge Integration for Health (CIDACS), Oswaldo Cruz Foundation, Salvador, Bahia, Brazil; 2 Rondônia Oswaldo Cruz Foundatio, Oswaldo Cruz Foundation, Porto Velho, Rondônia, Brazil; 3 Federal Center for Technological Education of Minas Gerais, Belo Horizonte, Minas Gerais, Brazil; 4 Department of Statistics, Federal University of Bahia, Salvador, Bahia, Brazil; 5 Center of Mathematics of University of Porto (CMUP), University of Porto, Porto, Portugal

**Keywords:** COVID-19, epidemiology, public health, cohort study

## Abstract

**Objectives:**

To classify the most up-to-date factors associated with COVID-19 disease outcomes in Brazil.

**Design:**

Retrospective study.

**Setting:**

Nationwide Brazilian COVID-19 healthcare registers.

**Participants:**

We used healthcare data of individuals diagnosed with mild/moderate (n=70 056 602) or severe (n=2801 380) COVID-19 disease in Brazil between 26 February 2020 and 15 November 2021.

**Main outcome measures:**

Risk of hospitalisation and mortality affected by demographic, clinical and socioeconomic variables were estimated. The impacts of socioeconomic inequalities on vaccination rates, cases and deaths were also evaluated.

**Results:**

15.6 million SARS-CoV-2 infection cases and 584 761 COVID-19-related deaths occurred in Brazil between 26 February 2020 and 15 November 2021. Overall, men presented a higher odds of death than women (OR=1.14, 95% CI 1.13 to 1.15), but postpartum patients admitted to hospital wards were at increased odds of dying (OR=1.23, 95% CI 1.13 to 1.34) compared with individuals without reported comorbidities. Death in younger age groups was notably higher in most deprived municipalities and also among individuals <40 years belonging to indigenous backgrounds compared with white patients, as shown by descriptive analysis. Ethnic/racial backgrounds exhibited a continuum of decreasing survival chances of mixed-race (OR=1.11, 95% CI 1.10 to 1.12), black (OR=1.34, 95% CI 1.32 to 1.36) and indigenous (OR=1.42, 95% CI 1.31 to 1.54) individuals, while those in most deprived municipalities also presented an increased odds of death (OR=1.38, 95% CI 1.36 to 1.40). Deprivation levels also affect the prompt referral of patients to adequate care. Our results show that the odds of death of individuals hospitalised for less than 4 days is more than double that of patients with close-to-average hospital stays (OR=2.07, 95% CI 2.05 to 2.10). Finally, negative vaccination status also increased the odds of dying from the disease (OR=1.29, 95% CI 1.28 to 1.31).

**Conclusions:**

The data provide evidence that the patterns of COVID-19 mortality in Brazil are influenced by both individual-level health and social risk factors, as well as municipality-level deprivation. In addition, these data suggest that there may be inequalities in the timely provision of appropriate healthcare that are related to municipality-level deprivation.

**WHAT IS ALREADY KNOWN ON THIS TOPIC**

- Brazil is still confronted with sparce literature dedicated to studies describing a population-wide clinical and epidemiological profile of COVID-19.
- Most works were targeted at specific populations (eg, ~SARS-CoV-2 infections among individuals with diabetes or cancer), specific Brazilian territories (eg, ~the Amazonic region) or regarding only initial waves of the epidemic in the country.

**WHAT THIS STUDY ADDS**

- Considering the fast-changing scenario associated with COVID-19 disease, many open questions remain on this subject, and further analyses using the most comprehensive data sets to date, as we have performed here, are essential for the management and reduction of the burden caused by COVID-19, as well as the direct and indirect effects on socioeconomic conditions.
- We gathered, to the best of our knowledge, the most comprehensive data set on COVID-19 hospitalisations in Brazil, comprising 18 months of observations since the onset of COVID-19.
- By considering vaccination status and socioeconomic deprivation levels, in addition to demographic factors, we uncovered how socioeconomic inequalities compounded the severity of the pandemic, creating additional risk factors beyond the natural history of the disease, shifting mortality to younger people in poor zones, posing a severe threat to puerperal women, and decreasing case ascertainment among the lower socioeconomic strata.

**HOW THIS STUDY MIGHT AFFECT RESEARCH, PRACTICE OR POLICY**

- Our results pinpoint the need for equity on health access in this and similar countries, ultimately revealing that the burden of COVID-19 and future pandemics can only be mitigated with equitable health resource allocation and reduction of economic asymmetries.

## Introduction

 Since the WHO declared pandemic status for COVID-19—caused by SARS-CoV-2—on 11 March 2020, more than 604 million cases have been reported worldwide, resulting in more than 6.5 million deaths (by 2 September 2022).^1 2^ These estimates represent a lower bound of case ascertainment, as the under-reporting of COVID-19 cases and deaths has been a recurrent problem in many countries.^1^

Brazil, one of the world’s largest and most populated nations, has tallied over 34.4 million COVID-19 cases and 684 000 deaths in the same period, ranking third and second globally in terms of numbers of cases and deaths, respectively. Substantial interest has been devoted to exploring the epidemiological factors underlying cases, hospitalisation events and deaths associated with COVID-19, as pinpointing these elements can better help define effective policies to be directed at priority groups in order to prevent severe outcomes of the disease. While individual traits including older age, sex (male), the presence of one or more comorbidities, and, more recently, negative vaccination status, have all been identified as risk factors for severe COVID-19 outcomes, the roles played by specific socioeconomic factors, such as healthcare access and quality, are less understood.^3 4^

By leveraging the availability of national-scale data distributed across multiple administrative database systems, we performed a retrospective analysis aimed at identifying the factors underlying severe COVID-19 outcomes (hospitalisations and deaths) in Brazil. Key aspects related to social inequalities were identified as drivers of mortality in this highly unequal middle-income country and may have shaped the course of this disease in other countries as well, revealing the added challenges that affected regions faced as COVID-19 spread, which has undoubtedly further heightened the gap between developed and developing nations.

## Methods

### Study design

The present study constitutes a retrospective analysis of reported SARS-CoV-2 infections in the Brazilian population. We used de-identified data on individuals to describe the clinical and epidemiological profiles of confirmed SARS-CoV-2 infections (ie, notified cases), COVID-19 hospital and intensive care unit (ICU) admissions, as well as inform risk stratification assessments associated with mortality.

Each SARS-CoV-2 case was classified in accordance with the individual’s clinical condition: mild/moderate or severe/critical infection. The criteria for classifying infected individuals with SARS-CoV-2 as having mild/moderate or severe/critical disease are given elsewhere.^5 6^ Additionally, information on patient age, sex, race/ethnicity, reported comorbidities and geographical location was obtained. For patients requiring hospitalisation, vaccination status was reported, as well as duration from onset of symptoms to hospital admission, and length of stay in hospital ward or ICU setting until the time of outcome (ie, death or discharge). Socioeconomic condition was also aggregated into the analysis via a local deprivation index (detailed in Data sources) associated with each patient’s municipality of residence. Furthermore, by applying data on vaccination coverage throughout the country, we offer an exploratory analysis in an attempt to help guide measures aimed at prioritising intervention in vulnerable regions.

### Data sources

We used de-identified, national-scale data on SARS-CoV-2 infections and vaccination status. The syndromic flu database SF*db*), maintained by the Secretariat of Health Surveillance, Brazilian Ministry of Health (SVS/MS), contains data on mild/moderate suspected cases of COVID-19.^5^ Confirmed COVID-19 cases in SF*db* that progress to severe/critical status are then entered in the severe acute respiratory disease database (SARD*db*), which contains notifications of all severe/critical cases of COVID-19 requiring hospitalisation, including those not necessarily previously reported inSF*db*. A schematic diagram detailing the patient selection process using these data sets is shown in online supplemental figure 1. It is important to note that in SF*db* there are cases (8.9%) with no reported symptoms (eg, individuals tested during sanitary barriers established in some airports; or because of a family member testing positive; due to occupational reasons, among others). Therefore, the descriptive analysis of SF*db* cases in our work also includes reported asymptomatic infections.

Following the start of the vaccination campaign in Brazil, patient records in SARD*db* also included information on vaccination status (vaccinated/not-vaccinated). To perform vaccine coverage analysis, information on vaccinated individuals, regardless of infection status, was obtained as a separate, individualised and de-identified data set. All data related to SARS-CoV-2 infections and vaccination status were obtained from the SVS/MS and the National Immunization Program Information System.^6 7^ The national COVID-19 immunisation programme started on 18 January 2021,^8^ such that the period comprised in this analysis spans less than 10 months since its launch, when most individuals had one of the following vaccination status: (1) Partially vaccinated: individuals that had taken the first of a two-dose vaccine; (2) Fully vaccinated: individuals that had taken two vaccine doses or one dose of the single-shot Ad26.COV2.S (Johnson & Johnson) vaccine; or (3) Boosted: individuals that had taken two vaccine doses (or one dose of the single-shot Ad26.COV2.S (Johnson & Johnson) vaccine), plus a booster shot.

Lastly, each individual was associated with a local index value to evaluate the effect of socioeconomic inequality based on the Brazilian Deprivation Index (BDI), a multivariable localised deprivation index, developed and calculated for each Brazilian municipality by the Center for Data and Knowledge Integration for Health.^9^

BDI measures, calculated using data from the 2010 Brazilian census, represent the results of a combination of municipal-level z-scores derived from the percentage of families with per capita income below half of the Brazilian monthly minimum wage (510 Brazilian Reals in 2010, ~US$290 at the time);^9^ the percentage of illiteracy in individuals aged 7 years or older; and the percentage of people without adequate access to potable water, sanitation systems, regular garbage collection, bathroom or shower, and toilet. The resulting metric incorporates these factors and ranks municipalities on a scale of most deprived (BDI=2.73) to least deprived (BDI=−1.76). The index follows an exponential distribution, thus, values close to zero represent average deprived municipalities, while relatively few municipalities represent elevated levels of deprivation.^9^ In addition, to assess the effect of BDI on the severity of COVID-19, we considered the distribution of cases in SARD*db* by BDI municipality scores. Therefore, considering the quantiles of this distribution, we established five classes: Class 0 (least deprived), corresponding to municipalities with BDI values falling in (−1.76 to –1.42); Class 1, values in (−1.42 to –1.33); Class 2, values in (−1.33 to –1.08); Class 3, values in (−1.08 to –0.63); and Class 4, values in (−0.63 to 2.73) (most deprived).

All data collection and preprocessing were performed using the Platform for Analytical Models in Epidemiology (PAMEpi).^10^ All coding and analyses were performed in Python/PySpark language using Pandas, NumPy, SciPy and statsmodels packages, and a code repository is freely available on GitHub.^11^ Our data are publicly available and can be downloaded following reference Pereira et al.^12^

### Statistical analyses

We first performed a descriptive analysis to detail the distribution of reported cases of SARS-CoV-2 infection in accordance with demographic factors (sex, age, race/ethnicity), comorbidities, BDI index class and vaccination status. Our analysis was then expanded to investigate the duration of time from symptom onset through the length of hospital stay (inpatient ward or ICU) until discharge/death.

Our descriptive analysis and the resulting disease profile was used to identify risk factors associated with severe outcomes. These risk factors were then employed to estimate the odds of death by COVID-19. A logistic regression model was used to estimate the outcomes of death (1) or survival (0), considering the following categorical variables (ie, risk factors): age: 0–17 years, 18–29 years, 30–39 years, 40–49 years, 50–64 years (reference), 65–74 years, 75–84 years and 85+ years; sex: male or female (reference); Brazilian Institute of Geography and Statistics ethnic/racial classification: white (reference), black, mixed-race, Asian and Indigenous; deprivation index values (BDI) (reference: Class 0 (least deprived)); ICU admission (reference: no); vaccinated with at least one dose or unvaccinated (reference: vaccinated with at least one dose); length of hospitalisation (inpatient ward or ICU): 0–4 days, 5–11 days (reference), 12–40 days and 40+days; reported comorbidities (lung disease, immunodeficiency, obesity, Down syndrome, kidney disease, chronic neurological disease, postpartum, chronic haematological disease, diabetes, asthma, heart disease, liver disease (reference: no reported comorbidity)). Descriptive analysis, ORs and 95% CIs were calculated and provided as online supplemental data. Importantly, considering that population data are employed in the current analysis, CIs are provided exclusively as an additional descriptive tool; therefore, statistical significance was not evaluated for any of the described parameters.

## Results

### Profile of SARS-CoV-2 infections registered in Brazil

We conducted a nationwide retrospective analysis of SARS-CoV-2 infections registered in two Brazilian administrative databases: SF*db*, which we used to extract mild/moderate cases, and SARD*db*, which covers severe cases of infection requiring hospitalisation. The derived data set comprised 20 months of registered events beginning with the onset of SARS-CoV-2 in Brazil, from 26 February 2020, until 15 November 2021.

In total, 15 512 543 clinically confirmed or laboratory-confirmed SARS-CoV-2 infections were identified, the majority (89.6%) of which were considered mild/moderate (registered in SF*db*); of these, 150 660 evolved to severe disease requiring hospitalisation (ie, first registered in SF*db* and subsequently recorded in SARD*db*). Considering cases that required immediate hospital admission, 1609 489 cases (10.4% of all confirmed infections) were reported directly to SARD*db*. In all, 584 761 (3.8% of 15 512 543) fatalities occurred during the studied period, of which 119 345 (20.4% of 584 761) ensued from originally mild/moderate cases. We can observe that 44.4% of the total fatalities (registered in SARD*db*) occurred following hospital admission to inpatient wards, while 55.6% were recorded post-ICU admission. Online supplemental figure 1 details the process employed to record notifications of SARS-CoV-2 infections, classified as mild/moderate or severe for the purposes of this study.

Table 1 presents an overview of the disease profile inferred from case notification data, stratified according to demographic, clinical and socioeconomic characteristics. Considering all notified cases, a higher proportion occurred among adults aged 18–64 years (80.3%), with women corresponding to 52.5% of all cases. The racial/ethnic background of infected individuals was primarily white (36.6%) and mixed-race (36.1%). Most cases did not report comorbidities (84.7%). Similar proportions were observed when analysing only the mild/moderate cases reported in SF*db*; however, considering those leading to severe outcomes registered in SARD*db*, substantial differences were noticed. An increasing tendency towards severe infection was observed in older age groups, with individuals aged 50–74 years representing 48.6% of cases; men predominated (55.7%), and 59.9% reported at least one comorbidity. In particular, heart disease (31.1%), diabetes (22.3%) and obesity (8.3%) were the most commonly reported conditions.

**Table 1:** Epidemiological profile of reported SARS-CoV-2 infections in Brazil

Characteristics	AllNumber (%)	Mild/moderate infectionsNumber (%)	Severe infectionsNumber (%)
**Demographic**			
Sex			
Female	8223 086 (52.50%)	7443 644 (53.54%)	779 442 (44.28%)
Male	7434 608 (47.47%)	6454 170 (46.42%)	980 438 (55.7%)
Missing	5509 (0.04%)	5240 (0.04%)	269 (0.02%)
Age			
0–17 years	1166 434 (7.45%)	1137 821 (8.18%)	28 613 (1.63%)
18–29 years	2971 044 (18.97%)	2898 372 (20.85%)	72 672 (4.13%)
30–39 years	3344 955 (21.36%)	3156 881 (22.71%)	188 074 (10.69%)
40–49 years	3072 708 (19.62%)	2789 866 (20.07%)	282 842 (16.07%)
50–64 years	3186 945 (20.35%)	2656 512 (19.11%)	530 433 (30.14%)
65–74 years	1131 459 (7.22%)	805 792 (5.80%)	325 667 (18.5%)
75–84 years	556 319 (3.55%)	334 430 (2.41%)	221 889 (12.61%)
85+ years	233 240 (1.49%)	123 284 (0.89%)	109 956 (6.25%)
Missing	99 (<0.01%)	96 (<0.01%)	3 (<0.01%)
Ethnic/racial background			
White	5728 461 (36.57%)	4988 434 (35.88%)	740 027 (42.04%)
Black	601 678 (3.84%)	526 013 (3.78%)	75 665 (4.3%)
Mixed-race	5651 508 (36.08%)	5057 329 (36.38%)	594 179 (33.76%)
Asian	558 317 (3.56%)	540 941 (3.89%)	17 376 (0.99%)
Indigenous	54 590 (0.35%)	51 100 (0.37%)	3490 (0.2%)
Missing	3068 649 (19.59%)	2739 237 (19.70%)	329 412 (18.72%)
**Comorbidities**			
No reported comorbidity	13 269 270 (84.72%)	12 562 815* (90.36%)	706 455 (40.14%)
Lung disease	289 469 (1.85%)	237 617† (1.71%)	51 852 (2.95%)
Immunodeficiency	119 501 (0.76%)	83 881 (0.60%)	35 620 (2.02%)
Obesity	234 173 (1.50%)	88 455 (0.64%)	145 718 (8.28%)
Down syndrome	4551 (0.03%)	–	4551 (0.26%)
Kidney disease	101 185 (0.65%)	45 829 (0.33%)	55 356 (3.14%)
Neurological chronic disease	54 523 (0.35%)	–	54 523 (3.10%)
Diabetes	798 612 (5.10%)	406 126 (2.92%)	392 486 (22.30%)
Haematological chronic disease	10 472 (0.07%)	–	10 472 (0.59%)
Asthma	42 118 (0.27%)	–	42 118 (2.39%)
Liver disease	12 694 (0.08%)	–	12 694 (0.72%)
Heart disease	1219 441 (7.79%)	672 099 (4.83%)	547 342 (31.10%)
Postpartum	8288 (0.05%)	3776 (0.03%)	4512 (0.26%)
Other	532 102 (3.40%)	82 607 (0.59%)	449 495 (25.54%)
**Deprivation index (BDI**)			
Class 0 (least deprived)	2529 659 (16.15%)	2188 639 (15.74%)	341 020 (19.37%)
Class 1	2236 972 (14.28%)	1891 569 (13.61%)	345 403 (19.62%)
Class 2	2610 579 (16.67%)	2264 361 (16.29%)	346 218 (19.67%)
Class 3	2615 743 (16.70%)	2271 437 (16.34%)	344 306 (19.56%)
Class 4 (most deprived)	4374 143 (27.93%)	4029 781 (28.98%)	344 362 (19.56%)
Missing	1296 107 (8.27%)	1257 267 (9.04%)	38 840 (2.21%)

* Includes missing data.

† SF*db* makes no distinction among respiratory diseases.

BDIBrazilian Deprivation IndexSFdbsyndromic influenza database

We then examined the relationship between socioeconomic conditions and COVID-19 prevalence. This analysis revealed substantial proportions of municipalities with high deprivation levels in mild/moderate cases reported in SF*db*, whereas cases were almost evenly distributed across BDI classes in SARD*db*. COVID-19 incidence per 100 000 inhabitants was higher in less deprived municipalities in both databases (online supplemental figure 2). Although this profile appears to underscore the lack of association between BDI variable and risk of contracting COVID-19, this analysis does not accurately depict the complex interplay between poverty and exposure to SARS-CoV-2. In particular, timely access to healthcare facilities and the quality of medical treatment, which affect disease severity and mortality, are not fully captured by the BDI.

### Profile of COVID-19-associated hospitalisation events

We then examined patterns in COVID-19-associated hospitalisation events, particularly the duration from the onset of symptoms to hospital admission and length of hospital stay. Hospitalisation outcomes were split into death or discharge. Differences in these variables among patients admitted to hospital wards or ICUs were also investigated.

**Figure 1:**
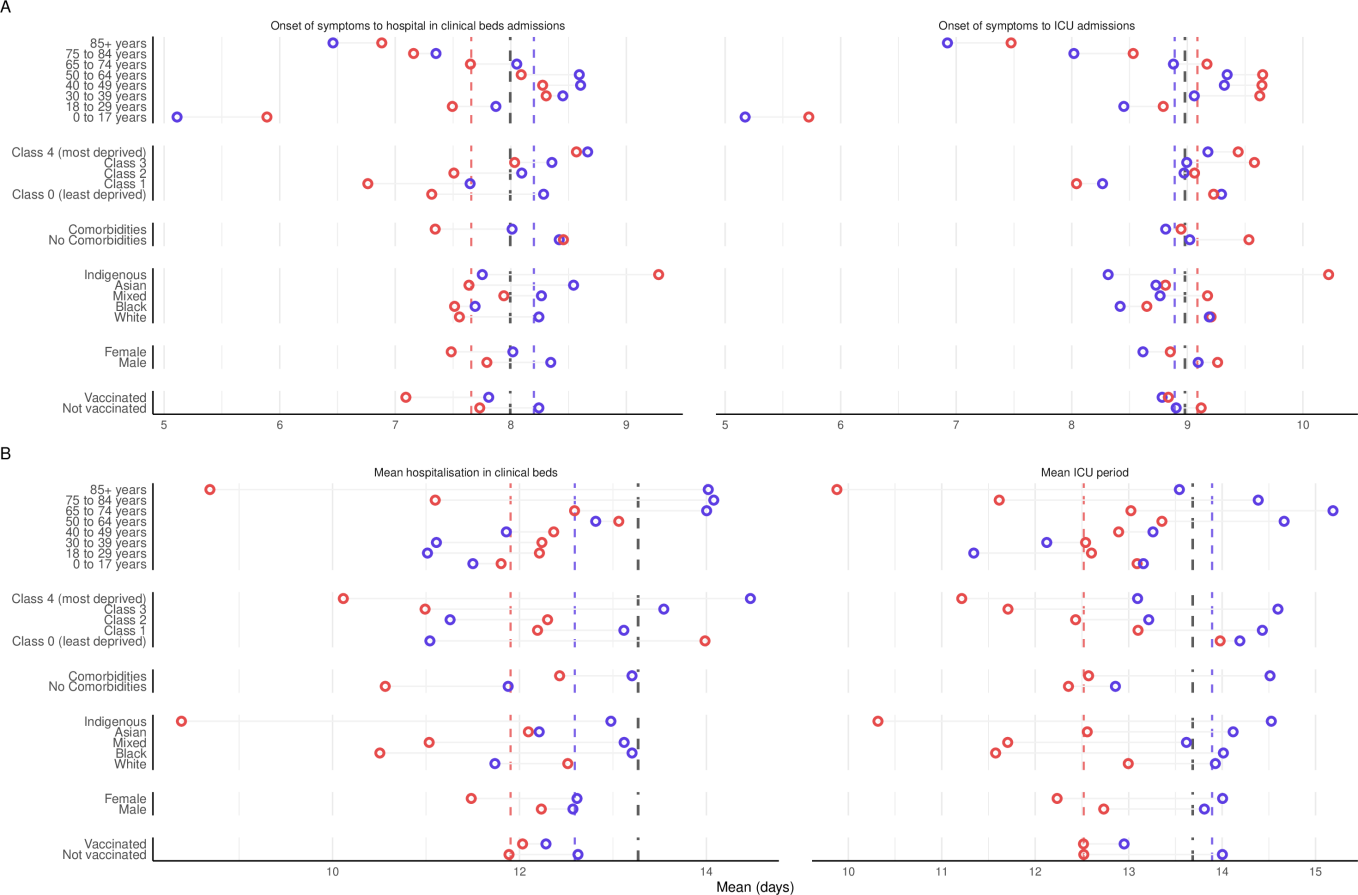
COVID-19 patient profile: Access to healthcare facilities and length of hospitalisation. (**A**) Mean period from onset of symptoms to hospital admission (hospital ward or ICU setting). (**B**) Mean duration of hospitalisation (hospital ward or ICU setting). Red and blue dots represent non-survivors and survivors, respectively. Dashed vertical lines indicate overall averages as follows: All cases (black), survivors (blue) and non-survivors (red). ICU, intensive care unit.

On average, the time from the onset of symptoms to ICU admission was slightly longer (9 days) than to hospital wards (8 days) (figure 1A). The length of stay in either setting was not considerably different between survivors and non-survivors. Non-survivors were admitted nominally earlier to hospital wards (7.7 days) than survivors (8.2 days). In contrast, survivors were admitted earlier to ICU facilities (8.9 days) compared with non-survivors (9.1 days). Although seemingly contradictory, this finding likely reflects periods of scarcity in specialised care facilities during surges of severe cases as a result of different waves of infection.

Our analysis then focused on factors influencing time from the onset of symptoms to hospitalisation. We observed that admissions to clinical wards occurred earlier at the extremes of the age groups (ranges: 0–17 years and 85+ years). Individuals with comorbidities also sought medical assistance earlier than those not reporting other illnesses. Vaccinated individuals, women and those living in municipalities with low deprivation (classes 0–2) also presented a shorter span between symptom onset and hospital admission compared with the average length of time. In contrast, the time to admission for patients from regions with high levels of deprivation (classes 3 and 4), those belonging to indigenous groups, and individuals aged from 40 years to 64 years were all longer than average (figure 1B). These discrepancies are also evident in the duration between onset of symptoms to ICU admission.

After patients were admitted to a healthcare facility, either a clinical ward or an ICU, the duration of hospitalisation played a critical role in survival. The length of hospital stay for patients admitted to wards or ICUs averaged, respectively, 12.3 days and 13.1 days (figure 1B).

Patients living in municipalities within BDI Class 0 and Class 2 who recovered after admission to clinical wards had shorter hospital stays (11.1 days) than those with fatal outcomes (13.1 days) (figure 1B). Similar patterns were observed for patients of white race/ethnicity (11.7 days for survivors, 12.5 days for non-survivors); and those aged 0–49 years (11.4 days for survivors, 12.1 for non-survivors). Patients aged 18–39 years admitted to ICU also presented similar profiles, with survivors staying on average 11.7 days while non-survivors remained an average of 12.6 days. A reversed pattern was seen for patients in wards, where the average length of hospitalisation for non-survivors was shorter than that of survivors in more deprived municipalities: Class 4 (average hospital stay for survivors: 14.5 days; 10.1 for non-survivors) and Class 3 (survivors: 13.5 days; 11 for non-survivors). Similar trends were noted for individuals of indigenous ethnic/racial (survivors: 13 days; 8.4 for non-survivors), mixed-race (survivors: 13.1 days; 11 for non-survivors) and black (survivors: 13.2 days; 10.5 for non-survivors) backgrounds, as well as for patients aged 75+ years (survivors: 14 days; 9.9 for non-survivors).

Non-surviving patients presented a lower mean period in ICU (12.5 days) than those who survived (13.9 days). However, disparities were still evidenced as the mean length of hospital stay was much shorter in more deprived municipalities, BDI Classes 2, 3 and 4, as well as in patients of indigenous, mixed-race or black race/ethnicity, women, and patients aged 75+ years, in comparison to others from the least deprived municipalities (BDI Classes 0 and 1), white patients, Asian, men and other age groups.

**Figure 2:**
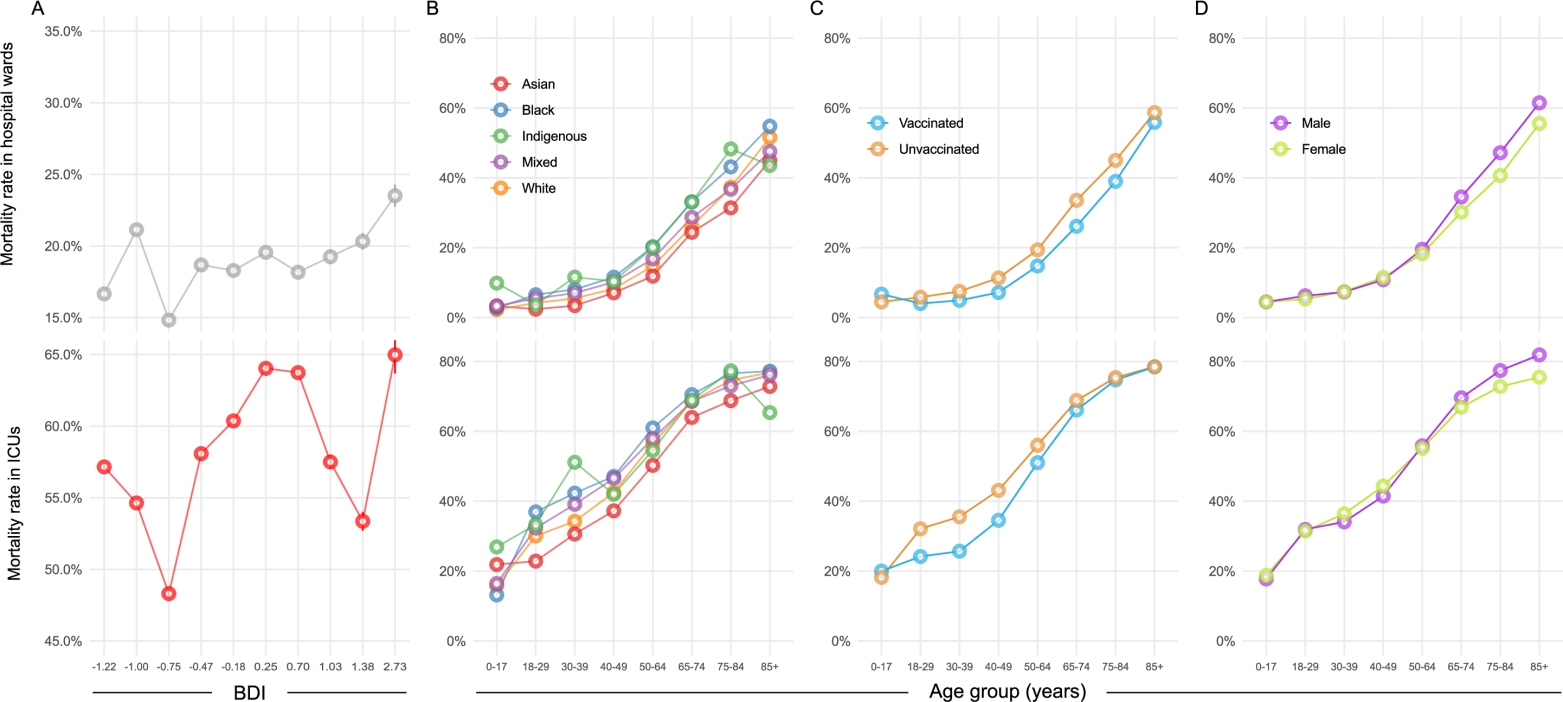
Mortality rates for severe cases of COVID-19 admitted to Brazilian hospital wards or ICUs, grouped according to (**A**) Deprivation class (BDI), (**B**) Ethnic/racial background, (**C**) Vaccination status and (**D**)Sex. BDI, Brazilian Deprivation Index; ICU, intensive care unit.

Overall, the mean period from the onset of symptoms to death was 19 days. Figure 2 shows mortality rates (MRs) in patients admitted to hospital wards or ICU facilities. MRs were observed to increase with older age irrespective of ethnic/racial background (figure 2B), vaccination status (figure 2C) or sex (figure 2D). In some cases, higher MRs were found among similarly aged individuals, for example, men aged 65+ years were more likely to die in hospital wards and ICUs compared with similarly aged women. Likewise, individuals of indigenous ancestry had higher MRs in both hospital settings compared with other ethnic/racial backgrounds across almost all age groups (figure 2B). Poverty was also a crucial factor influencing survival, as MRs were higher for patients living in more deprived regions, notably with respect to those admitted to hospital wards (figure 2A). Further analysis revealed higher mortality following hospitalisation in younger individuals residing in higher BDI municipalities (online supplemental figure 3).

Mortality was lower in vaccinated individuals across all age groups, irrespective of admission type (figure 2C). The effect of vaccine protection against death following ICU hospitalisation was more evident in patients aged 18–29 years, 30–39 years and 40–49 years, as evidenced by 24.7%, 27.7% and 19.9%, respectively, lower MRs in comparison to similarly aged unvaccinated individuals.

Table 2 compares the profiles of vaccinated and unvaccinated individuals in SARD*db* to those of all individuals in Brazil. Until 15 November 2021, 75.3% (161 029 567 individuals) of the population had been vaccinated, 24.1% of whom received the first dose (partially vaccinated individuals), while 64.2% were fully vaccinated (first and second doses of AZD1222/AstraZeneca, BNT162b2/Pfizer or CoronaVac/Butantan, or a single dose of Ad26.COV2.S/JJ), with 7.5% receiving a booster shot. Among infected individuals in SARD*db*, most individuals were fully vaccinated (55.9%), with doses administered between 2 months and 6 months prior to infection. However, the proportion of partially vaccinated among infected individuals (44.1%) was almost two times higher than that of the entire Brazilian population (24.1%).

**Table 2:** Vaccination profile in Brazil. Country-wide vaccination status compared with individuals with severe COVID-19 included in SARD*db* registries

Characteristics	All individuals vaccinatedNumber (%)	Vaccinated in SARD*db*			
		AllNumber (%)	SurvivorsNumber (%)	Non-survivorsNumber (%)	Mortalityrate
**Demographic**					
Sex					
Female	84 626 124 (52.55%)	87 287 (46.99%)	52 280 (47.54%)	30 873 (46.09%)	37.1%
Male	76 395 099 (47.44%)	98 450 (53.01%)	57 692 (52.46%)	36 109 (53.91%)	38.5%
Missing	8344 (0.01%)	–	–	–	–
Age					
0–17 years	13 455 036 (8.36%)	172 (0.09%)	139 (0.13%)	15 (0.02%)	9.7%
18–29 years	34 979 781 (21.72%)	3198 (1.72%)	2716 (2.47%)	281 (0.42%)	9.4%
30–39 years	30 769 890 (19.11%)	10 080 (5.43%)	8517 (7.74%)	1050 (1.57%)	11.0%
40–49 years	27 959 762 (17.36%)	18 680 (10.06%)	15 004 (13.64%)	2775 (4.14%)	15.6%
50–64 years	32 781 390 (20.36%)	46 692 (25.14%)	32 344 (29.41%)	12 161 (18.15%)	27.3%
65–74 years	12 991 054 (8.07%)	47 116 (25.37%)	26 247 (23.87%)	18 543 (27.68%)	41.4%
75–84 years	6054 621 (3.75%)	38 445 (20.70%)	17 522 (15.93%)	19 206 (28.67%)	52.3%
85+ years	2038 033 (1.27%)	21 360 (11.50%)	7485 (6.81%)	12 955 (19.34%)	63.4%
Ethnic/racial background					
White	55 316 895 (34.35%)	96 452 (51.93%)	56 103 (51.02%)	37 075 (55.35%)	39.8%
Black	7 064 407 (4.38%)	8142 (4.38%)	4346 (3.95%)	3394 (5.07%)	43.9%
Mixed-race	34 277 206 (21.28%)	52 983 (28.53%)	31 879 (28.99%)	18 454 (27.55%)	36.7%
Asian	*	1718 (0.92%)	1071 (0.97%)	575 (0.86%)	34.9%
Indigenous	413 981 (0.26%)	270 (0.15%)	156 (0.14%)	92 (0.16%)	37.1%
Missing	39 692 746 (25%)	26 178 (14.09%)	16 419 (14,93%)	7396 (11,01%)	31.1%
**Deprivation index (BDI**)					
Class 0 (least deprived)	23 982 198 (14.89%)	46 843 (25.22%)	28 382 (25.81%)	17 368 (25.93%)	38.0%
Class 1	26 940 761 (16.73%)	39 087 (21.04%)	21 834 (19.85%)	14 041 (20.96%)	39.1%
Class 2	27 681 992 (17.19%)	39 335 (21.18%)	24 207 (22.01%)	13 785 (20.58%)	36.3%
Class 3	30 930 189 (19.20%)	30 338 (16.33%)	17 855 (16.24%)	11 173 (16.68%)	38.5%
Class 4 (most deprived)	50 613 345 (31.43%)	26 700 (14.38%)	15 416 (14.02%)	9575 (14.29%)	38.3%
**Vaccine type**					
AZD1222/AstraZeneca	56 950 790 (35.37%)	64 942 (34.96%)	41 398 (37.64%)	20 467 (30.55%)	33.1%
CoronaVac/Sinovac	32 713 052 (20.31%)	83 787 (45.11%)	43 145 (39.23%)	36 754 (54.87%)	46.0%
BNT162b2/Pfizer	64 183 910 (39.86%)	10 155 (5.47%)	8227 (7.48%)	1444 (2.16%)	14.9%
Ad26.COV2.S/Johnson&Johnson	473 978 (0.29%)	1966 (1.06%)	1652 (1.50%)	222 (0.33%)	11.8%
**Vaccine status**					
Partially vaccinated (P)	38 782 441 (24.08%)	81 942 (44.12%)	46 633 (42.40%)	30 954 (46.21%)	39.9%
Fully vaccinated (F)	103 404 949 (64.21%)	103 801 (55.89%)	63 341 (57.60%)	36 032 (53.79%)	36.3%
Boosted (B)	12 134 340 (7.54%)	–	–	–	–
**Vaccine delay**					
P≥6 months	2221 822 (1.38%)	714 (0.38%)	305 (0.28%)	337 (0.50%)	52.5%
P between 2 months and 6 months	26 098 747 (16.21%)	14 899 (8.02%)	8558 (7.78%)	5562 (8.30%)	39.4%
P<2 months	10 701 657 (6.65%)	59 956 (32.28%)	34 398 (31.28%)	23 377 (34.90%)	40.5%
F≥6 months	9259 839 (5.75%)	5691 (3.06%)	2980 (2.71%)	2248 (3.36%)	43.0%
F between 2 months and 6 months	51 761 004 (32.14%)	39 161 (21.08%)	19 482 (17.72%)	17 283 (25.80%)	47.0%
F<2 months	42 144 324 (26.17%)	25 460 (13.71%)	13 230 (12.03%)	11 089 (16.55%)	45.6%
B>6 months	933 (<0.01%)	–	–	–	–
B between 2 months and 6 months	338 453 (0.21%)	–	–	–	–
B<2 months	11 794 954 (7.32%)	–	–	–	–

* Considerations are given in online supplemental note 1.

BDIBrazilian Deprivation IndexSARDdbsevere acute respiratory disease database

Concerning individuals who had received at least one dose in the vaccination campaign, the data revealed higher vaccine coverage among women (81.4%) than men (73.4%), with individuals aged 30–39 years exhibiting the higher vaccination rates (93.9)%. As expected, younger individuals aged 0–17 years presented the lowest vaccine coverage (28.7%). More than 80% of the remaining age groups had received at least one dose. Considering the ethnic/racial backgrounds of vaccinated individuals, 54.1% were white, 43.4% black, 37.1% mixed-race and 45.1% were indigenous. Inconsistencies were identified in data pertaining to Asian individuals, as detailed in online supplemental note 1. Information on ethnic/racial background was missing for 18.5% of the vaccinated individuals. With respect to socioeconomic conditions, vaccine administration was observed to decrease in municipalities with higher deprivation, as 78.1% of vaccinated individuals lived in BDI Class 0 (least deprived), 80.1% in Class 1, 78.7% in Class 2, 76.3% in Class 3 and 69.1% in Class 4 (most deprived) municipalities.

### Risk factors contributing to death following COVID-19

**Figure 3:**
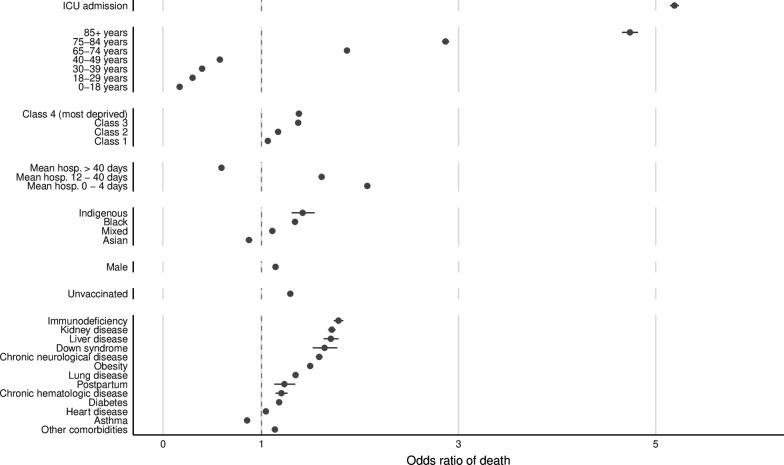
Risk factors contributing to death following hospitalisation by COVID-19 in Brazil. ORs compared with the reference of each group (see Methods) are shown on a log scale. Error bars represent the limits of the 95% CI for the OR. ICU, intensive care unit.

Next, we applied multivariate logistic regression using the SARD*db* registry to investigate patient risk factors with regard to the odds of death following hospitalisation due to COVID-19 (figure 3).

Compared with patients in clinical wards, an over five times higher odds of death was estimated for individuals requiring ICU admission (OR=5.19, 95% CI 5.14 to 5.24). Men died at a 1.14 (95% CI 1.13 to 1.15) higher odds than women. Odds of mortality also increased with age. Compared with the reference age group (50–64 years), OR ranged from 0.17 (95% CI 0.16 to 0.18) for those aged 0–17 years to 4.74 (95% CI 4.66 to 4.82) for individuals 85 years or older. Individuals living in less deprived municipalities (BDI Classes 0 and 1) exhibited similar odds of death. However, a 1.38 higher odds of death (95% CI 1.36 to 1.40) was seen in the most impoverished municipalities (BDI Classes 3 and 4). In addition, the chance of death in non-vaccinated individuals was 1.29 (95% CI 1.28 to 1.31) times higher than in those who were vaccinated with at least one dose.

Differences in ethnic/racial background were also associated with odds of death. Mixed-race (OR=1.11, 95% CI 1.10 to 1.12), black (OR=1.34, 95% CI 1.32 to 1.36) and indigenous (OR=1.42, 95% CI 1.31 to 1.54) groups were more likely to die than white individuals, while an Asian background appeared to be protective (OR=0.87, 95% CI 0.84 to 0.91).

Comorbidities were also identified as contributing to an increased chance of death, with a 50% greater odds of dying linked to liver disease, immunodeficiency, kidney disease, Down syndrome, chronic neurological disease, and obesity compared with the absence of a reported comorbidity. Postpartum women also had an augmented chance of death (OR=1.23, 95% CI 1.13 to 1.34). This last result reveals that although men present overall higher chances of death compared with women, when observing the OR for postpartum women an inverse pattern is seen.

Interestingly, patients with heart disease showed no differences compared with those with no reported comorbidities (OR=1), while the OR of death in individuals with asthma was decreased (OR=0.85, 95% CI 0.83 to 0.88).

We observed an association between the length of hospitalisation stay and mortality (online supplemental figure 5). To incorporate this variable into the regression model, hospital stays were discretised into four classes: <4 days, 4–11 days (reference group), 12–40 days and 40+ days.

Patients hospitalised for 40+ days were more likely to survive compared with close-to-average hospitalisation periods of 4–11 days (OR=0.59, 95% CI 0.58 to 0.61). On the other hand, patients treated for less than 4 days died twice (OR=2.0, 95% CI 2.05 to 2.09) as frequently as those in the reference group. Patients hospitalised between 12 days and 40 days were also more likely to die (OR=1.60, 95% CI 1.59 to 1.62). These results suggest a more severe clinical condition in patients with shorter hospital stays (<4 days), while patients hospitalised for 12 days to 40 days may have had a more progressive worsening of their condition resulting in death or improvement leading to recovery.

Individuals living in lowest deprivation municipalities, BDI Classes 0 and 1, were equally likely to die. However, the most impoverished municipalities (BDI Classes 3 and 4) had 1.38 higher odds of death (95% CI 1.36 to 1.40). Finally, non-vaccinated individuals also had an increased chance of death (OR=1.29, 95% CI 1.28 to 1.31).

### Analysis of the impact of missing ethnic/racial data on the regression model results

Racial/ethnic information has revealed an important factor contributing to an increase in OR mortalities as outlined previously. However, the corresponding impact of missing data in this variable (corresponding to 18.72% of registries in SARD*db*, see table 1) needs to be assessed. In online supplemental note 2, we present the results of a sensitivity analysis^13^,^15^ assessing the impact of missing data in the race/ethnicity variable on the outputs of the regression model. The results confirm the robustness of the analysis and, despite the percentage of missing data, the interpretation of the outcomes remained unaltered.

## Discussion

By leveraging national-scale data on hospitalisations and notifications of syndromic influenza across two Brazilian administrative databases harbouring over 15 million registries, we aimed to understand how individual risk factors and socioeconomic elements influenced the outcomes of severe COVID-19 in the country.

We found that mild/moderate cases prevailed in younger adults, women and those with no comorbidities, while severe cases were associated with older age, male sex and those with pre-existing health conditions. In addition, incorporating variables related to hospitalisation profile, ethnic/racial background, levels of deprivation and vaccination status added further layers to our analysis by enabling an improved interpretation of the outcomes of the pandemic in the country.

### Characteristics of severe COVID-19 in Brazil: influence of comorbidities and socioethnic/racial factors

We then examined the risk factors contributing to death following severe COVID-19. Male sex, age over 65 years, at least one reported comorbidity, hospitalisation profile, negative vaccination status, and place of residence in highly deprived municipalities all independently warranted increased chances of death. Ethnic/racial backgrounds associated with black, mixed-race and indigenous groups emerged as contributory factors to severe outcomes as well.

Consistent with global evidence, a higher proportion of men were associated with severe cases, also presenting increased in-hospital mortality.^16^ However, our analysis revealed a slightly increased mortality of women aged 30–49 years in ICUs compared with men of the same age group. In particular, the chances that postpartum women have of dying from the disease was higher than that of men or individuals with comorbidities such as diabetes or chronic haematological disease. SARS-CoV-2 infection has been associated with an increased risk of maternal mortality or major morbidity outcomes due to obstetric complications in pregnancy and postpartum women.^17 18^ Studies conducted in Brazil have also evidenced the harmful effects of COVID-19 on maternal health,^19^,^22^ now supported by our population-scale study.

Multiple comorbidities registered in the studied databases were found to increase the odds of death. Immunodeficiencies, kidney disease, liver disease, Down syndrome, chronic neurological disease, obesity, lung disease, chronic haematological disease and diabetes were all independently associated with augmented odds of dying. Notably, we found no significant increase in the odds of death in patients with asthma, in agreement with previous works,^23^,^25^ including the recent meta-analysis by Sunjaya *et al* that pooled 51 studies and identified comparable risk ratios of hospitalisation (RR=1.18, 95% CI 0.98 to 1.42) and mortality (RR=0.94, 95% CI 0.76 to 1.17) in these patients compared with patients without asthma.

Mortality increased significantly in an age-dependent manner, and the interplay among age, ethnic/racial background and socioeconomic factors revealed the added challenges that underprivileged groups endured as COVID-19 spread in Brazil. Particularly, mortality among indigenous children and those aged 30–39 was pronounced compared with other ethnic/racial backgrounds. A similar trend, but among black and mixed-race individuals, was seen by Baqui *et al*, who explored 11 321 hospitalisation events during earlier stages of the pandemic, which these authors referred to as a race/ethnicity effect.^26^ Ours and other^23^ studies that used more comprehensive data sets strengthen this finding. By incorporating socioeconomic elements in our examination through the lens of the BDI,^9^ we uncovered the negative consequences that COVID-19 effected on poorer populations in Brazil. Individuals living in municipalities of lower socioeconomic strata presented decreased access to vaccines, delayed admission to hospitalisation, and increased chances of death compared with individuals of less deprived municipalities. The finding that non-surviving patients in municipalities with high levels of deprivation (BDI Classes 3 and 4) presented lower than average hospitalisation lengths suggests an already deteriorated health condition on admittance for many of these hospitalisation events, as early access to specialised healthcare has shown to be critical for the successful management of severe cases of COVID-19.^27^ On this basis, we argue that not only a race/ethnicity effect occurred in Brazil, but rather a socioethnic/racial effect that disproportionately impacted underprivileged groups and minorities leading to increased mortality due to COVID-19. Similar effects were also observed in high-income countries.^28 29^

Here we punctuated how vulnerable populations in Brazil faced severe inequalities in accessing healthcare facilities and benefiting from appropriate treatment conditions during the COVID-19 pandemic. While persons with mild conditions such as cough and fatigue may recover at home, those with severe illnesses such as pneumonia and acute respiratory distress syndrome may require hospital admission for intensive care, which in turn is costly and demands infrastructure to provide treatment and guarantee access for all patients. In sum, our retrospective, large-scale study contributes to assessing the risk of severe COVID-19 in the country and pinpointing where efforts to reduce morbidity and mortality should be directed. Suppressing incidence among priority groups is key to achieving COVID-19 control and averting more deaths,^30^,^32^ and should also be emphasised in preparation for future pandemics.

### Limitations

Limitations of the present study include the barrier to data integrating different healthcare-related systems in Brazil, most of which are usually not harmonised. National linking^33^ is a way to make the data more complete for detailed analyses, such as those related to maternal and child health and comorbidities (eg, asthma). The quality of some administrative databases is also a factor of concern. Our analysis revealed the main weaknesses in reporting information for the variables used in this work. Analysing the impact of missing information and even under-reporting are important key factors that can add value to these data sets. The sensitivity analysis performed (online supplemental note 2) helps to understand under what circumstances these anomalies in the data can affect the analysis presented in this work. Here we explored if missing race/ethnicity entries may affect the estimated mortality OR values. Results from this analysis indicate that this effect is not large enough to change the qualitative picture presented here. Further studies on under-reporting would bring a full picture of the pandemic impact on most deprived municipalities, a work that goes beyond the scope of this manuscript. Furthermore, the vaccination database was unreliable to explore the vaccine coverage of individuals from Asian backgrounds (see online supplemental note 1). This only affected the descriptive analysis (as shown in table 2), left with a lack of information for this category. Finally, socioeconomic inequalities could be further explored by considering the intramunicipality deprivation index, potentially providing a more refined assessment.

## Conclusions

By considering multiple dimensions of the hospitalisation data routinely collected in Brazil during the 18 months since onset of COVID-19, combined with vaccination status and socioeconomic deprivation levels, we uncovered how individual factors, hospitalisation profiles and socioeconomic inequalities compounded the severity of the pandemic, and created additional risk factors beyond the natural history of the disease, including a shift in mortality to younger people in more deprived regions, as well as posing a severe threat to postpartum women. Historically underprivileged populations, including the indigenous, as well as those in low-income municipalities, with more limited access to specialised healthcare, were also at increased risk of severe disease, ultimately revealing that the burden of COVID-19 and future pandemics can only be mitigated with equitable health resource allocation and reduction of socioeconomic asymmetries.

## supplementary material

10.1136/bmjgh-2022-009489 online supplemental file 1

## Data Availability

Data are available in a public, open access repository.
