# Supplementary material for: Profile of COVID-19 in Brazil—risk factors and socioeconomic vulnerability associated with disease outcome: retrospective analysis of population-based registers
Source: BMJ Glob Health. 2022 Dec 14;7(12):e009489. doi: 10.1136/bmjgh-2022-009489 (PMC9755904; doi:10.1136/bmjgh-2022-009489)
Supplement: online supplemental file 1 [file bmjgh-7-12-s001.pdf]

Supplementary Figures

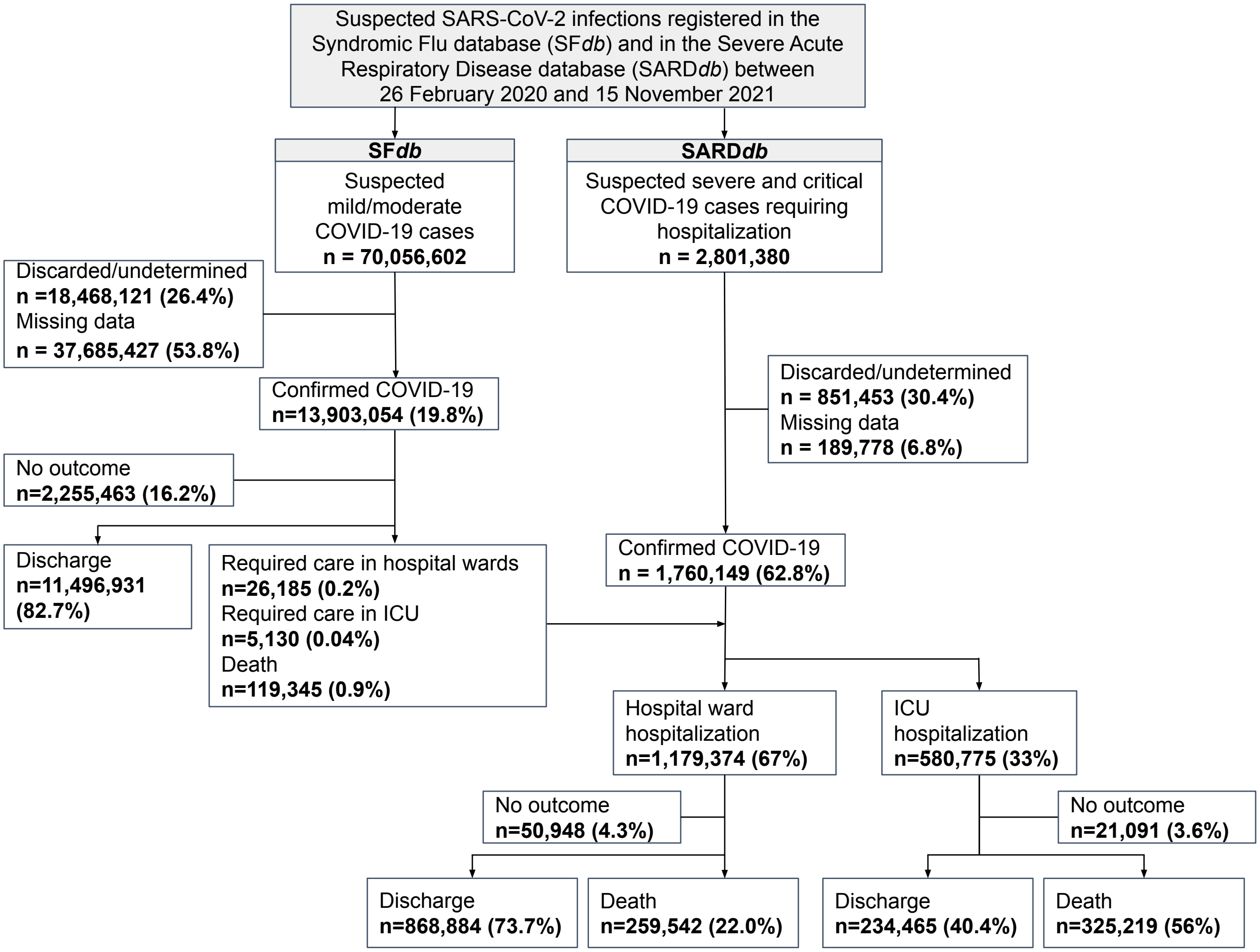

Supplementary Figure 1. Recording of mild/moderate and severe cases of SARS-CoV-2 infections.

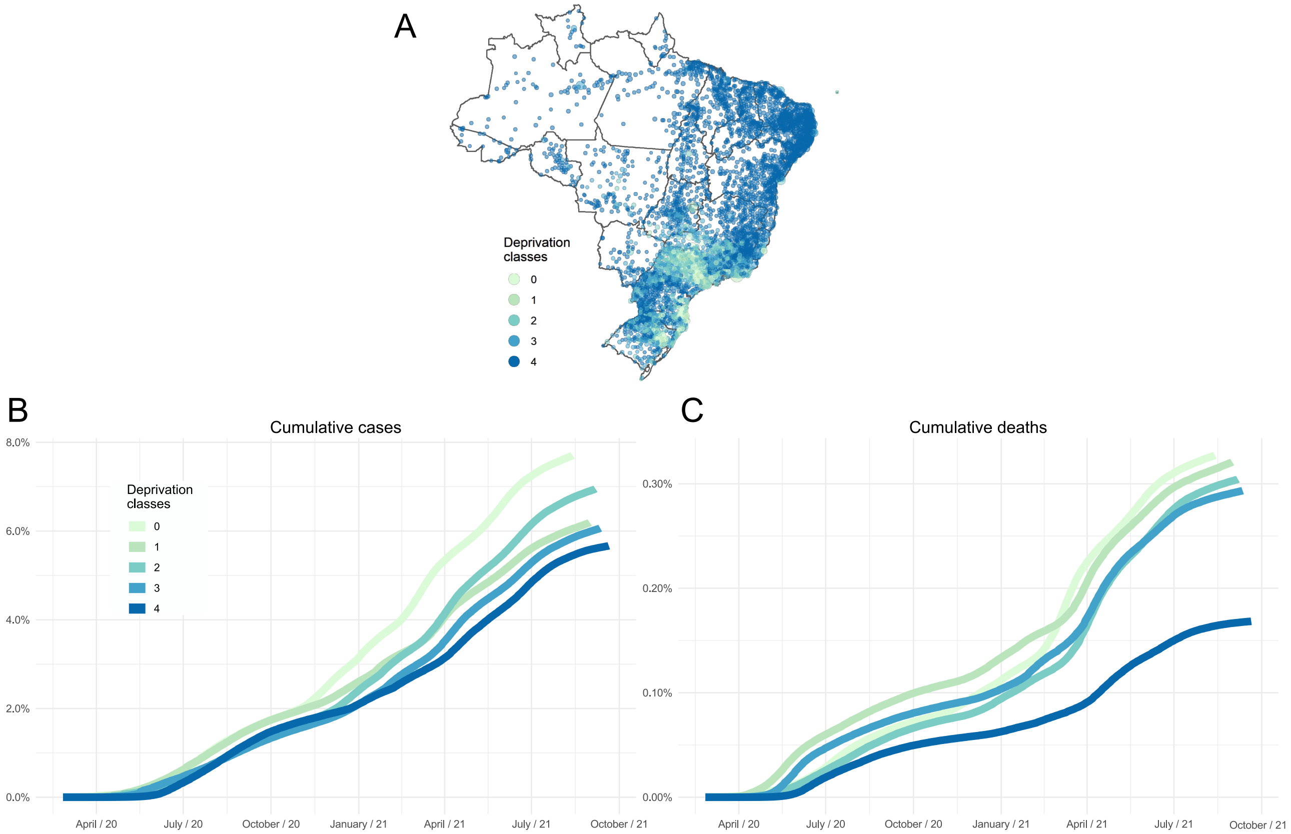

Supplementary Figure 2. (**A**) Brazilian municipalities according to BDI classes and observed cumulative series of cases (**B**) and deaths (**C**) within each class up to 15 November 2021.

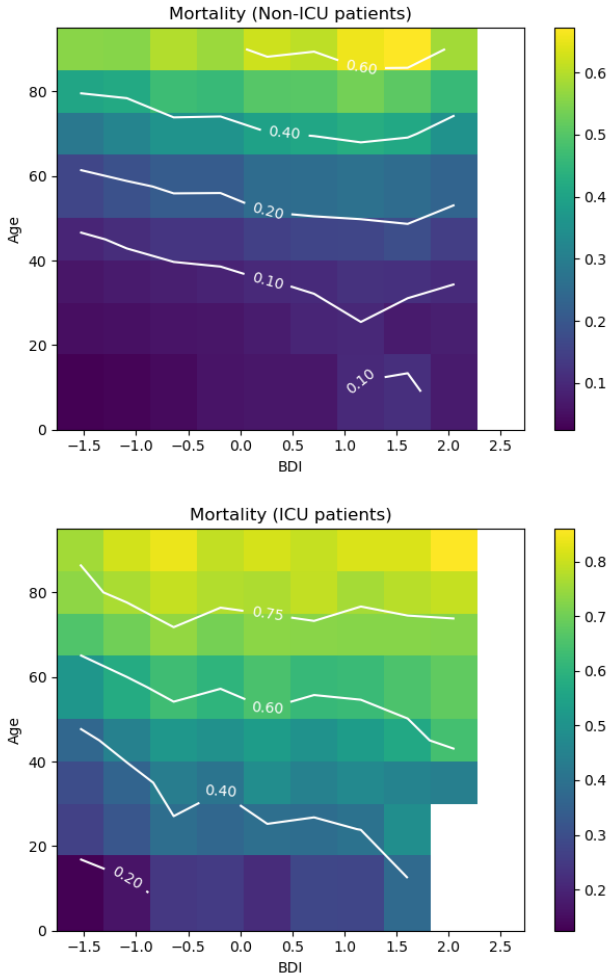

Supplementary Figure 3. Contour plot of mortality rates in Brazilian hospital wards and ICU facilities according to age and deprivation areas (BDI). White tiles represent BDI×age overlaps having less than 100 reported cases, which were not used to estimate mortality.

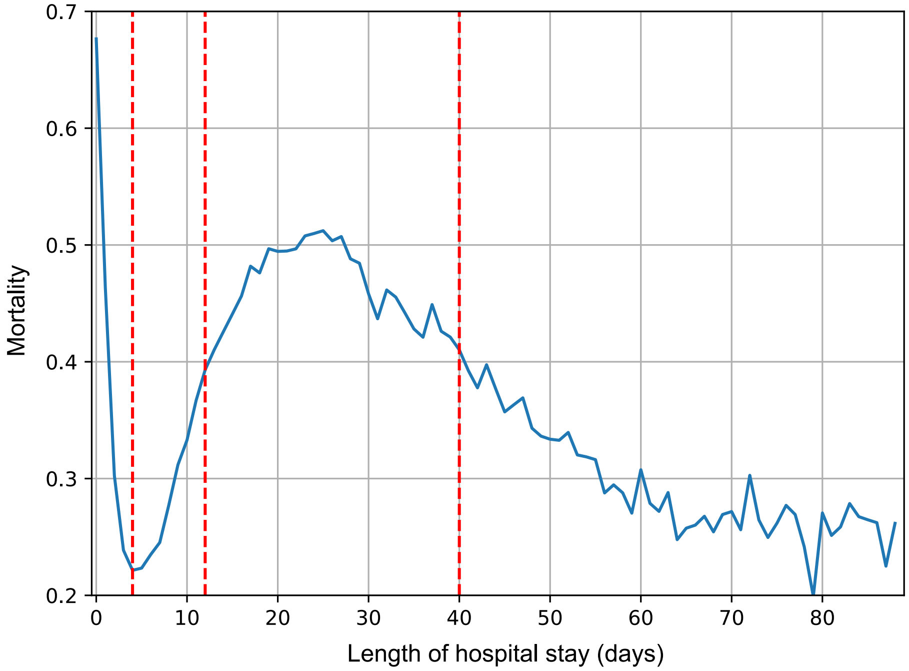

Supplementary Figure 4. Relationship between length of hospital stay and mortality rates. The dashed vertical lines indicates the limits of the four groups used during the regression analysis after discretization: <4 days, 4-11 days (reference group), 12-40 days, and 40+ days.

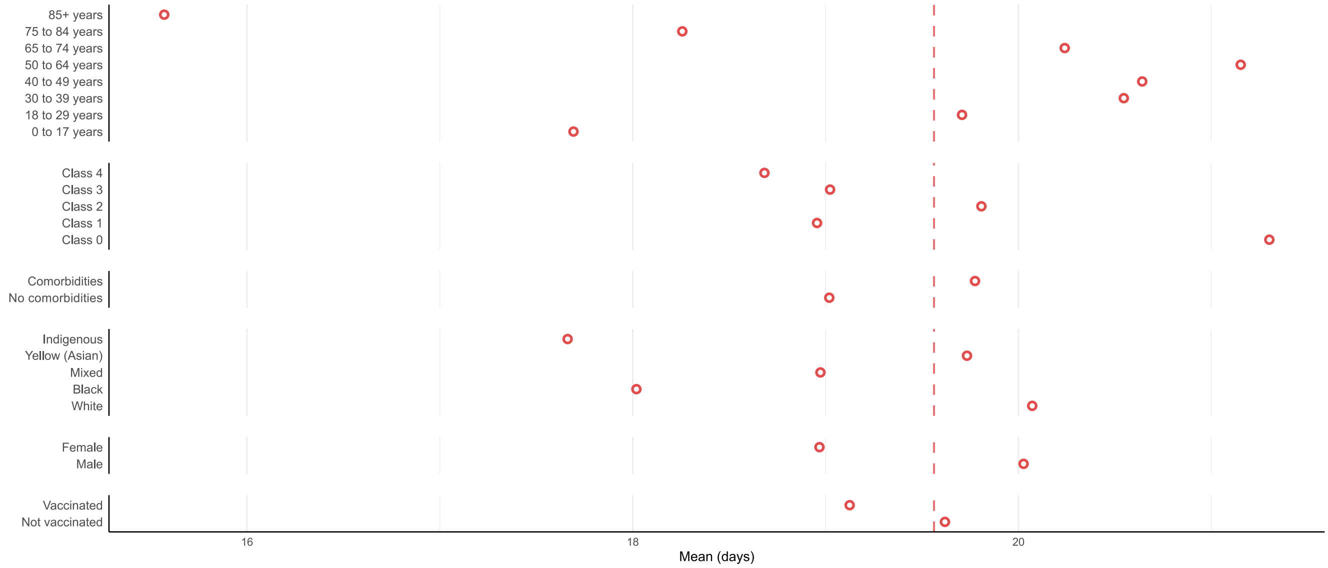

Supplementary Figure 5. Time span from onset of symptoms to death of COVID-19 patients in Brazil. The dashed vertical line indicates the overall mean.

Supplementary Tables

Characteristics	Odds ratio	IC (95%)
**Demographic**		
Sex		
Female	Reference	–
Male	1.14	[1.13, 1.15]
Age		
0-17 years	0.17	[0.16, 0.18]
18-29 years	0.30	[0.29, 0.31]
30-39 years	0.398	[0.391, 0.404]
40-49 years	0.58	[0.57, 0.59]
50-64 years	Reference	–
65-74 years	1.87	[1.85, 1.89]
75-84 years	2.87	[2.83, 2.90]
85+ years	4.74	[4.66, 4.82]
Ethnic/racial background		
White	Reference	–
Black	1.34	[1.32, 1.36]
Mixed-race	1.11	[1.10, 1.12]
Asian	0.87	[0.84, 0.91]
Indigenous	1.42	[1.31, 1.54]
**Deprivation index (BDI)**		
Class 0 (least deprived)	Reference	–
Class 1	1.06	[1.05, 1.08]
Class 2	1.17	[1.15, 1.18]
Class 3	1.37	[1.35, 1.39]
Class 4 (most deprived)	1.38	[1.36, 1.40]
**Patient's characteristics**		
Not vaccinated	1.29	[1.28, 1.31]
Lung disease	1.35	[1.32, 1.38]
Immunodeficiency	1.78	[1.73, 1.83]
Obesity	1.49	[1.47, 1.52]
Down syndrome	1.64	[1.52, 1.77]
Kidney disease	1.71	[1.68, 1.75]
Chronic neurological disease	1.59	[1.55, 1.62]
Diabetes	1.18	[1.17, 1.19]
Puerperal	1.23	[1.13, 1.34]
Chronic hematologic disease	1.20	[1.14, 1.26]
Asthma	0.85	[0.83, 0.88]
Liver disease	1.70	[1.63, 1.78]
Heart disease	1.04	[1.04, 1.05]
Other comorbidities	1.14	[1.13, 1.15]
**Hospitalization profile**		
Needed ICU	5.19	[5.14, 5.24]
Length of hospital stay		
0–3 days	2.07	[2.05, 2.10]
4–11 days	Reference	–
12–39 days	1.61	[1.60, 1.63]
$\geq$ 40 days	0.59	[0.58, 0.61]

Supplementary Table 1. Results of the logistic regression for the mortality probability in SARD*db* registries.

**Characteristics**	Unvaccinated in SARD*db*			
	All	Survivors	Non-survivors	Mortality
**Demographic**				
Sex				
Female	692,155 (43.96%)	438,574 (44.15%)	225,580 (43.57%)	34.0%
Male	881,988 (56.02%)	554,644 (55.83%)	292,127 (56.42%)	34.5%
Age				
0-17 years	28,441 (1.81%)	24,086 (2.42%)	2,076 (0.40%)	7.9%
18-29 years	69,474 (4.41%)	58,161 (5.85%)	7,845 (1.52%)	11.9%
30-39 years	177,994 (11.31%)	144,930 (14.59%)	25,278 (4.88%)	14.9%
40-49 years	264,162 (16.78%)	201,106 (20.24%)	51,945 (10.03%)	20.5%
50-64 years	483,741 (30.73%)	317,362 (31.95%)	147,114 (28.41%)	31.7%
65-74 years	278,551 (17.69%)	142,061 (14.30%)	126,359 (24.40%)	47.1%
75-84 years	183,444 (11.65%)	76,390 (7.69%)	100,638 (19.44%)	56.8%
85+ years	88,596 (5.63%)	29,276 (2.95%)	56,520 (10.92%)	65.9%
Ethnic/racial background				
White	643,575 (40.88%)	414,717 (41.75%)	211,745 (40.90%)	33.8%
Black	67,523 (4.29%)	38,228 (3.85%)	26,454 (5.11%)	40.9%
Mixed-race	541,196 (34.37%)	327,539 (32.97%)	189,284 (36.56%)	36.6%
Yellow (Asian)	15,658 (0.99%)	10,068 (1.01%)	4,947 (0.96%)	32.9%
Indigenous	3,220 (0.20%)	1,931 (0.19%)	1,185 (0.23%)	38.0%
Missing	303,234 (19.26%)	200,892 (20.22%)	84,160 (16.25%)	29.5%
**Deprivation index (BDI)**				
Class 0 (least deprived)	294,177 (18.68%)	202,898 (20.43%)	86,477 (16.70%)	29.9%
Class 1	306,316 (19.46%)	196,092 (19.74%)	98,317 (18.99%)	33.4%
Class 2	306,883 (19.49%)	198,421 (19.97%)	96,756 (18.69%)	32.8%
Class 3	313,968 (19.94%)	188,308 (18.96%)	110,867 (21.41%)	37.1%
Class 4 (most deprived)	317,662 (20.18%)	182,408 (18.36%)	116,090 (22.42%)	38.9%

Supplementary Table 2. Profile of unvaccinated individuals with severe COVID-19 included in SARD*db* registries.

Supplementary Notes

Note 1: Identification of ethnic/racial backgrounds in Brazil

Brazil has a wide variety of race/ethnic groups, with individuals originating from different regions in the world. The Brazilian census surveys the race/ethnicity of the Brazilian population based on self-declaration. That is, individuals are asked about their ethnic/racial background according to the following options: White, indigenous, black, Asian, and "Pardo" (mixed-race). Based on 2010 census, white represented 47.7% of the population, black 7.6%, yellow 1.1%, mixed 43.1% and indigenous 0.4%. According to a more recent data from the National Household Sample Survey (PNAD) in year 2019, these proportions were maintained, with 42.7% of Brazilians self-referred as white, 46.8% as mixed-race, 9.4% as black, and 1.1% as yellow (Asian) or indigenous[1]. In the presence of a healthcare worker, individuals will generally be associated with an race/ethnic group without being asked. Therefore, it can be misleading and generate errors on a posterior analysis involving such a variable. However, it is important to consider this variable in studies, since several results in the literature have already displayed the influence of race/ethnicity on socioeconomic and health factors.

Considering the datasets in our work, we observed that descriptive counts on the vaccination dataset for the race/ethnic group variable is compromised. The vaccination dataset counts each dose applied as a line. That is, if a person receives two shots, this information will be registered twice in the dataset, corresponding to two lines of data. Since individual identifications are not automatically stored or updated from one system, a person may be identified as white when the first dose was administered and black at the moment of the second shot.

Accordingly, 161,029,567 individuals were registered in the vaccination dataset (accessed on 15 November, 2021). Around 0.32% (521,966) of these were identified as being from two or more ethnic/racial background, therefore, we regarded these individuals as "mixed-race". Additionally, there were cases when a person was identified as belonging to an ethnic/racial background on one occasion when given a vaccine, while no information was noted when another shot was given. In these cases, we considered the ethnic group reported on any of these occasions and defined then as probably assigned to an ethnic/racial background (these cases are denoted as Probably Black/Asian/White/Indigenous/or Mixed-race in Supplementary Table 3).

Asian background is the only group to present a drastic change according to the proportions of the population established in Brazil by IBGE. The number of Asian people in the vaccine dataset represents already over 1,000% of the Asian population estimated in 2021 (Supplementary Table 3). There are documentations[2] that these populations increased its population by 173% in the 2010 national census. Some hypotheses attribute this to an increase in the migration of Asians to Brazil or that there was an increase in the reassertion of this particular race/ethnic identity, as has occurred among mixed-race and black individuals recently. Therefore, this increase may be still reflected in the census of 2020.

Category	Number of vaccinated in VACdb (% relative to total vaccinated)	% of the population vaccinated
Total	161,029,567	75.25% of Brazilian population (213,971,284) in 2021.
Total White	55,316,895 (34.35%)	54.15% of White population (102,145,244).
White<br>Probable White	55,083,531 (34.20%)<br>233,364 (0.14%)	
Total Black	7,064,407 (4.38%)	43.37% of Black population (16,286,808).
Black<br>Probable Black	7,040,405 (4.37%)<br>233,364 (0.02%)	
Total Mixed	34,277,206 (21.28%)	37.13% of Mixed population (92,301,882).
Mixed<br>Probable Mixed	34,056,795 (21.14%)<br>220,411 (0.13%)	
Total Indigenous	413,981 (0.26%)	45.11% of Indigenous population (917,622).
Indigenous<br>Probable Indigenous	413,367 (0.26%)<br>614 (<0.01%)	
Total Yellow (Asian)	23,742,366 (14.74%)	**1015.39% of Asian population (2,338,235).**
Asian<br>Probable Asian	23,725,521 (14.73%)<br>16,845 (0.01%)	
Unknown	39,692,746 (25%)	18.55% of Brazilian population.

Supplementary Table 3. Proportion of vaccinated people in Brazil according to ethnic/racial background.

Note 2: Analysis of the impact of missing ethnic/racial data on the regression model results

In order to classify and rank the most current factors related to outcomes of COVID-19 disease in Brazil, individual race/ethnicity information was available and considered in the study. However, in Table 1 (main manuscript) it can be seen that a total of 3,068,649 (19.59%) of the reported cases in SF*db* and SARD*db* do not contain any race/ethnicity information. Among these, 329,412 (18.72%) cases are from SARD*db*, a large percentage that may impact the analysis performed in our work. Motivated by this, we performed a sensitivity analysis to assess whether the missing data in the race/ethnicity variable would result in changes in the patterns observed in the outputs of the regression model. Details of the methodology used during this analysis can be found in Papageorgiou et al.[4], Eekhout et al.[5] and Pedersen et al.[6].

Distribution of race/ethnicity missing values

Our first step was to analyze whether the missing data on the race/ethnicity variable are associated with the other covariates considered in our regression analysis. In Figure 6 we plot the proportion of cases for each covariate included in the regression model (age, sex, BDI classes, ICU admission, vaccination status, length of hospitalisation stay and reported comorbidities). Note that, a total of 1,760,149 individuals (rows) are considered in the analysis (Supplementary Figure 11), where 1,430,737 of those individuals have race/ethnicity information provided and 329,412 have missing data.

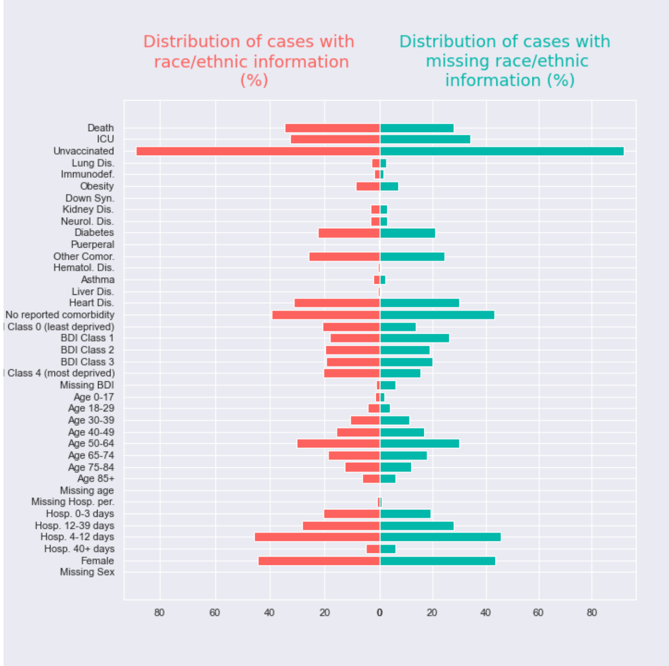

Supplementary Figure 6. Distribution of cases with and without race/ethnic information among categories of the variables age, sex, BDI classes, ICU admission, vaccination status, length of hospitalisation stay and reported comorbidities. Data from the SARD*db* from 26 February 2020, until 15 November 2021.

We found that the distribution of cases with and without the race/ethnic information is similar among categories of all variables. A slight variation in this distribution is more noticeable across the categories of variables ICU and BDI. The proportion of cases requiring ICU with missing race/ethnicity information is higher compared to cases with race/ethnicity reported. With respect to the classes of variable BDI, the distribution is more uniform for cases in which information for race/ethnicity is present, whereas a greater proportion is noticed for cases in municipalities within BDI Class 1 whenever race/ethnicity is missing.

Effect of the missing values on the Logistic Regression results

Figure 6 also indicates that the missing race/ethnicity data can be Missing Completely at Random, i.e., observations have equal probability to be missing. However, the slight variations in missing data proportions found in the ICU and BDI variables, indicate a more realistic scenario when the data may be Missing at Random, i.e., the probability of a missing value depends on other observed variables[4]. In this case, to carry out the sensitivity analysis it is necessary to perform multiple imputation of

the data, that is, to reproduce multiple copies of the original incomplete dataset in which the missing values in the race/ethnic variable are substituted with randomly-chosen values selected conditionally on the other covariates. In our analysis we assume the missing data depends on the ICU, BDI and age variables, the latter due to its high influence on the disease outcome.

Next, the model is fitted to each imputed dataset. Thereafter, an estimate of the parameters is obtained by averaging the results obtained from each analysis, and the associated standard errors are calculated according to Rubin's rule[4].

Additionally to the previous sensitivity analysis, we also considered the two following cases: an univariate analysis accounting for the impact of only the race/ethnicity variable on disease outcome, and a second model with all covariates, except the race/ethnicity variable. Results for all new fitted models are discussed in the following section.

Results

The multiple imputation procedure was done with 18 copies of the database, and results are presented in Supplementary Table 4. For most of the variables, the multiple imputation returned odds ratios (OR) that are compatible with the complete case analysis described in the main manuscript. The exceptions are the outcome OR for some ethnic/racial backgrounds (Black and Mixed-race), BDI Class 1, and ICU requirements variable. While statistically significant, these differences do not affect the conclusions of the manuscript, as Mixed-race and Black individuals have increased odds of death in both analysis.

The two additional analyses performed returned similar results: the racial/ethnic background univariate logistic regression (Supplementary Table 4) has results that are similar to the multiple imputation approach for all racial/ethnic groups except indigenous, which may be due the large difference of the population pyramid in this population group[3]. The other logistic regression that used all variables but racial/ethnic background (Supplementary Table 4) also shows similar results to the complete case and multiple imputation approaches, with small differences on the BDI variable. In this analysis, the OR of the most deprived municipalities (BDI Class 4) is higher than in other cases.

	**Complete-case**		**Multiple Imputation**		**Univariate analysis**		**Complete case without race/ethnicity**	
**Characteristics**	Odds ratio	IC (95%)	Odds ratio	IC (95%)	Odds ratio	IC (95%)	Odds ratio	IC (95%)
**Demographic**								
Sex								
Female	Reference	–	–	–			–	–
Male	1.14	[1.13, 1.15]	1.14	[1.13, 1.15]			1.14	[1.13, 1.15]
Age								
0-17 years	0.17	[0.16, 0.18]	0.17	[0.16, 0.18]			0.17	[0.16, 0.18]
18-29 years	0.30	[0.29, 0.31]	0.31	[0.30, 0.31]			0.31	[0.30, 0.31]
30-39 years	0.40	[0.39, 0.40]	0.40	[0.39, 0.40]			0.40	[0.39, 0.40]
40-49 years	0.58	[0.57, 0.59]	0.58	[0.57, 0.58]			0.58	[0.57, 0.58]
50-64 years	Reference	–	–	–			–	–
65-74 years	1.87	[1.85, 1.89]	1.87	[1.85, 1.89]			1.86	[1.85, 1.88]
75-84 years	2.87	[2.83, 2.90]	2.86	[2.82, 2.89]			2.84	[2.81, 2.88]
85+ years	4.74	[4.66, 4.82]	4.62	[4.55, 4.69]			4.59	[4.52, 4.66]
Ethnic/racial background								
White	Reference	–	–	–	–	–		
Black	1.34	[1.32, 1.36]	1.28	[1.25, 1.30]	1.28	[1.26, 1.30]		
Mixed-race	1.11	[1.10, 1.12]	1.09	[1.08, 1.10]	1.07	[1.06, 1.07]		
Asian	0.87	[0.84, 0.91]	0.89	[0.86, 0.93]	0.93	[0.90, 0.96]		
Indigenous	1.42	[1.31, 1.54]	1.34	[1.24, 1.45]	1.13	[1.05, 1.21]		
**Deprivation index (BDI)**								
Class 0 (least deprived)	Reference	–	–	–			–	–
Class 1	1.06	[1.05, 1.08]	0.97	[0.96, 0.98]			0.99	[0.98, 1.00]
Class 2	1.17	[1.15, 1.18]	1.15	[1.13, 1.16]			1.16	[1.15, 1.18]
Class 3	1.37	[1.35, 1.39]	1.35	[1.34, 1.37]			1.39	[1.38, 1.41]
Class 4 (most deprived)	1.38	[1.36, 1.40]	1.39	[1.38, 1.41]			1.46	[1.44, 1.48]
**Patient's characteristics**								
Not vaccinated	1.30	[1.28, 1.31]	1.30	[1.28, 1.31]			1.30	[1.29, 1.32]
Lung disease	1.35	[1.32, 1.38]	1.34	[1.31, 1.37]			1.34	[1.31, 1.37]
Immunodeficiency	1.78	[1.73, 1.83]	1.79	[1.74, 1.83]			1.79	[1.74, 1.83]
Obesity	1.49	[1.47, 1.52]	1.49	[1.47, 1.51]			1.48	[1.46, 1.50]
Down syndrome	1.64	[1.52, 1.77]	1.67	[1.55, 1.79]			1.66	[1.55, 1.78]
Kidney disease	1.71	[1.68, 1.75]	1.72	[1.69, 1.76]			1.73	[1.70, 1.77]
Chronic neurological disease	1.59	[1.55, 1.62]	1.57	[1.54, 1.61]			1.57	[1.54, 1.60]
Diabetes	1.18	[1.17, 1.19]	1.18	[1.17, 1.19]			1.18	[1.17, 1.19]
Puerperal	1.23	[1.13, 1.34]	1.25	[1.15, 1.35]			1.26	[1.16, 1.37]
Chronic hematologic disease	1.20	[1.14, 1.26]	1.23	[1.18, 1.29]			1.23	[1.17, 1.29]
Asthma	0.85	[0.83, 0.88]	0.85	[0.83, 0.88]			0.85	[0.83, 0.88]
Liver disease	1.70	[1.63, 1.78]	1.71	[1.64, 1.78]			1.71	[1.64, 1.78]
Heart disease	1.04	[1.04, 1.05]	1.03	[1.02, 1.04]			1.03	[1.02, 1.04]
Other comorbidities	1.14	[1.13, 1.15]	1.13	[1.12, 1.14]			1.13	[1.12, 1.14]
**Hospitalization profile**								
Needed ICU	5.19	[5.14, 5.24]	4.97	[4.94, 5.01]			4.97	[4.93, 5.01]
Length of hospital stay								
0–3 days	2.07	[2.05, 2.10]	2.06	[2.04, 2.08]			2.06	[2.04, 2.08]
4–11 days	Reference	–	–	–			–	–
12–39 days	1.61	[1.60, 1.63]	1.60	[1.59, 1.62]			1.60	[1.59, 1.62]
≥ 40 days	0.59	[0.58, 0.61]	0.59	[0.58, 0.60]			0.59	[0.58, 0.60]

Supplementary Table 4. Sensitivity analysis of the impact of missing ethnic/racial data on the logistic regression for the mortality probability in SARD*db* registries. Complete-case describe the results with all variables included as described in the main manuscript; Multiple imputation is the result as described in this note; Univariate analysis only accounts for race/ethnicity variable as covariable; and Complete case without race/ethnicity correspond to the analysis as described in main manuscript without race/ethnicity variable.

References

1. IBGE, https://educa.ibge.gov.br/jovens/conheca-o-brasil/populacao/18319-cor-ou-raca.html, Accessed 15 November, 2021.
2. O Globo, "Asian population increased by 173% in Brazil, according to the 2010 Census", http://oglobo.globo.com/pais/mat/2011/04/30/populacao-asiatica-aumentou-173-no-brasil-segundo-censo-de-2010-92asp. Accessed 15 November, 2021.
3. https://censos.ibge.gov.br/2013-agencia-de-noticias/releases/14389-asi-ibge-mapeia-a-populacao-indigena.html
4. Papageorgiou, Grigorios, et al. "Statistical primer: how to deal with missing data in scientific research?." Interactive cardiovascular and thoracic surgery 27.2 (2018): 153-158.
5. Eekhout, Iris, et al. "Missing data: a systematic review of how they are reported and handled." Epidemiology 23.5 (2012): 729-732.
6. Pedersen, Alma B., et al. "Missing data and multiple imputation in clinical epidemiological research." Clinical epidemiology 9 (2017): 157.
